# Effect of applying head-shaking maneuver before Epley maneuver in BPPV

**DOI:** 10.1016/j.bjorl.2020.12.015

**Published:** 2021-02-13

**Authors:** Suphi Bulğurcu, Eyup Baz, Selin Güleryüz, Evren Erkul, Engin Çekin

**Affiliations:** Sultan Abdulhamid Han Training and Research Hospital, Department of Otorhinolaryngology, İstanbul, Turkey

**Keywords:** Canalolithiasis, Dizziness, Otolith, Utricle, Vertigo

## Abstract

**Introduction:**

The Epley maneuver is applied in the treatment of benign paroxysmal positional vertigo, the BPPV. However, dizziness and balance problems do not improve immediately after the treatment.

**Objective:**

In this study, the effectiveness of the head-shaking maneuver before the Epley maneuver was investigated in the treatment of BPPV.

**Methods:**

Between March 2020 and August 2020, ninety-six patients with posterior semicircular canal BPPV were analyzed prospectively. The patients were divided into two groups: patients who underwent the Epley maneuver only in the treatment (Group 1) and patients who underwent the Epley maneuver after the head-shaking maneuver (Group 2). The results of the Berg balance scale and dizziness handicap index were evaluated before the treatment and at the first week after the treatment.

**Results:**

The improvement in functional, emotional, and physical dizziness handicap index and Berg balance scale values after the treatment was found to be statistically significant in both groups. It was determined that the change in functional and physical dizziness handicap index and Berg balance scale values of the patients in Group 2 was statistically higher than those in Group 1. Although, the change in emotional dizziness handicap index values in Group 2 was higher than those in Group 1, no statistical significance was found between the groups.

**Conclusion:**

As a result of our hypothesis, we think that in the treatment of posterior semicircular canal BPPV, the otoliths adhered to the canal can be mobilized by the head-shaking maneuver, and this will contribute to the increase of the effectiveness of the Epley maneuver.

## Introduction

Benign paroxysmal positional vertigo (BPPV), which was first identified by Barany, is the most common peripheral vestibular pathologic condition. BPPV is defined as an abnormal sensation of movement, usually evoked by sudden head movements. Typically, each vertigo attack lasts less than one minute. It has been reported in the literature that the prevalence of BPPV can vary between 10.7–64/100,000 and the lifetime prevalence can reach 2.1%. BPPV can occur at any age, however it is most frequently detected between the 5th and 7thdecades.^1^, ^2^ The specific cause of BPPV is still unknown, however it may be associated with various disorders of the inner ear or head trauma.^3^

Otoliths called autoconial debris that pass from the utricular macula to the semicircular canal are responsible for the pathogenesis of BPPV. This can happen in two different ways: canalolithiasis and cupulolithiasis. Canalolithiasis occurs when otoliths enter the canal from the non-bulbs end and move freely in the canal. Cupulolithiasis occurs by the adhesion of otoliths in the cupule of the crista ampullaris. The most commonly involved canal is the posterior semicircular canal and the form of posterior canal canalolithiasis is mostly observed.^4^

The diagnosis of BPPV is performed by using the Dix–Hallpike Maneuver (DM). Posterior canal canalolithiasis is diagnosed in patients with upward geotropic and tired nystagmus that lasts less than a minute, with latency duration of a few seconds with DM. Posterior canal cupulolithiasis is diagnosed in patients with DM with no latency and no fatigue by upward geotropic duration longer than one minute.^5^ Otolith repositioning maneuvers are applied to the patients diagnosed with canalolithiasis. Various treatment maneuvers are available in posterior canal BPPV. Hilton et al. in their review with 11 studies, they stated that the Epley Maneuver (EM) is a safe and effective treatment for posterior canal BPPV.^6^ The success rate of EM is ranged between 75%–89%.^7^

Kaplan et al. stated that the patients with BPPV negative were found to be BPPV positive as a result of the application DM with headshaking. The reason was the increase in the movement of adhering otoliths, and when DM was applied with head-shaking, the correct diagnosis rate increased by 14.8%.^8^ Ma et al. stated that applying DM after head-shaking maneuver will help to make an accurate diagnosis.^9^ Song et al. reported that in the lateral canal apogeotropic BPPV, otoliths on the utricular side would be released by head-shaking and then with barbecue maneuvers successfully treated.^10^ In the light of these studies, we hypothesized that applying head-shaking before EM in patients with posterior canal canalolithiasis may increase the success of EM. In this study, the results of Berg Balance Scale (BBS) and Dizziness Handicap Index (DHI) were evaluated in patients who underwent head-shaking maneuver before EM and who underwent EM without head-shaking maneuver before treatment and at the 1st week after treatment.

## Methods

Between March 2020 and August 2020, ninety-six patients diagnosed with posterior semicircular canal canalolithiasis were prospectively studied after obtaining local ethical approval (2020/27). Informed consent forms were signed by all patients.

Patients who were diagnosed with DM with posterior semicircular canal canalolithiasis were questioned about their age, gender, smoking and alcohol habits, comorbid disease, and whether they had been diagnosed with BPPV before. The patients were divided into two groups as EM applied without head-shaking maneuver (Group 1) and EM applied after head-shaking maneuver (Group 2). Patients were randomly assigned to either the Group 1 or Group 2 in the order of their enrollment in the study. The BBS and DHI values of the patients in both groups were examined before the treatment and at the first week of the treatment. DHI values were examined as subgroups as functional, emotional, and physical aspects. All assessments before the treatment and at the first week of the treatment were performed by one investigator. The investigator was blinded to which maneuver the patient received up to the point of data analysis.

### Head-shaking maneuver

The patient's head was brought to a 30° flexion position and eyes were left open. Then the maneuver was applied by the examiner by turning the patient's head horizontally at approximately 45° with a frequency of approximately 2–3 Hz for 10 s passively.

All patients were advised to rest after the treatment, no exercise was initiated, and no medical treatment was given.

Patients under 18 years of age, patients receiving medical treatment for vertigo, patients with cervical pathology, patients with hearing loss, patients with neurological disease, patients with multiple canal involvement, patients with subjective posterior BPPV, patients with cupulolithiasis and patients with anterior or lateral BPPV were excluded from the study.

### Statistical analysis

Power analysis was carried out before the study was designed; 48 patients were included in each of the groups, and a total of 96 patients were planned to be included in the study. Data were collected in Microsoft Excel format for the statistical analysis. Descriptive and analytical statistics were performed using the Statistical Package for the Social Sciences (SPSS) software version 11.0 (SPSS Inc., Chicago, IL, USA). In the analysis of continuous data, the normal distribution was found using the Kolmogorov–Smirnov test. Chi-Square test, Fisher's Exact Test, Mann Whitney *U* Test, and Wilcoxon test were carried out, as the data was suitable for the analysis for normal distribution. The results were evaluated at a 95% Confidence Interval and *p* < 0.05 as significance level.

## Results

Seventy female and twenty-six male patients with an average age of 47.20 ± 12.42 years (18–65 years of age) diagnosed with posterior semicircular canal canalolithiasis were studied. There are 48 patients in each group. No statistical difference was found between the two groups in terms of age, gender, comorbidities, smoking, alcohol habits, and in terms of having BPPV diagnosis previously (Table 1). In Group 1, it was found that functional, emotional, and physical DHI values of the patients statistically decreased after the treatment. In addition, it was found that the BBS values of the patients in Group 1 statistically increased after the treatment (Table 2). In Group 2, the functional, emotional, and physical DHI values of the patients statistically decreased after the treatment. In addition, it was found that the BBS values of the patients in Group 2 statistically increased after the treatment (Table 3). It was determined that the change in functional and physical DHI values and BBS values of the patients in Group 2 was statistically higher than in Group 1. Although, the change in emotional DHI values in Group 2 was higher than those in Group 1, no statistical significance was found between the groups (Table 4).

**Table 1:** Demographic characteristics of the patients.

	Group 1	Group 2	*p*
Gender (F/M)	38/10	32/16	0.17
Age	48.5 ± 12.63	45.91 ± 12.21	0.26
Comorbid disease			0.41
DM	13	11	
HT	13	5	
Lung disease	2	1	
CAD	2	3	
Smoking	12	15	0.49
Alcohol	5	5	–
Previously diagnosed BPPV	23	25	0.68

DM, Diabetes Mellitus; HT, Hypertension; CAD, Coronary Artery Disease.

**Table 2:** Group1 treatment evaluation.

	Functional DHI	Emotional DHI	Physical DHI	BBS
Pre-treatment	15.70 ± 9.52	8.45 ± 6.95	14.33 ± 5.70	50.95 ± 6.19
Post-treatment	5.04 ± 7.40	2.37 ± 4.78	4.00 ± 5.61	53.18 ± 4.88
*p*	<0.0001	<0.0001	<0.0001	<0.0001

BBS, Berg Balance Scale; DHI, Dizziness Handicap Index.

**Table 3:** Group 2 treatment evaluation.

	Functional DHI	Emotional DHI	Physical DHI	BBS
Pre-treatment	20.29 ± 9.82	9.95 ± 7.53	17.25 ± 6.59	50.06 ± 5.83
Post-treatment	4.41 ± 6.14	1.54 ± 2.83	4.04 ± 5.33	53.91 ± 3.25
*p*	< 0.0001	< 0.0001	< 0.0001	< 0.0001

BBS, Berg Balance Scale; DHI, Dizziness Handicap Index.

**Table 4:** Comparison of efficacy of Group 1 and Group 2 treatments.

	Functional DHI	Emotional DHI	Physical DHI	BBS
Group 1	10.62 ± 7.79	6.12 ± 6.06	10.12 ± 5.32	2.22 ± 3.16
Group 2	15.83 ± 9.16	8.41 ± 7.51	13.20 ± 6.62	3.85 ± 3.79
*p*	0.004	0.20	0.015	0.035

BBS, Berg Balance Scale; DHI, Dizziness Handicap Index.

## Discussion

BPPV is a common disorder and characterized by brief episodes of vertigo, unsteadiness and nausea. These symptoms are typically precipitated by a change in orientation of the head or body in relation to gravity. These positional changes often occur during common activities such as lying down in bed or reaching up to retrieve an object from a high shelf. Otoliths that pass from the utricular macula to the semicircular canal are responsible for the pathogenesis of BPPV. In most of the patients with BPPV the cause of the otoliths’ displacement is unknown. Otoliths’ displacement can be effectively treated with therapy maneuvers.^11^ Although, vertigo and nystagmus disappear after therapy maneuvers, patients often describe residual dizziness. Residual dizziness is defined as a feeling of heaviness in the head without vertigo and nystagmus, and a short-term sense of imbalance during standing and walking.^12^ In this respect, there are various hypotheses in the literature.^13^, ^14^, ^15^ As our hypothesis, otoliths adhering to the canal after EM may not settle into the utricle and create residual dizziness.

Epley stated that vibrations applied to the mastoid would be beneficial in order to increase the migration of the otoliths adhered to the canal.^16^ However, in studies conducted with a larger number of patient groups, it was found that there was no difference between otolith repository maneuvers and the vibrations.^17^, ^18^ Griech et al. stated that applying vibration to the mastoid can contribute positively in cases of persistent canalolithiasis.^19^ We believe that the movement of adhered otoliths can be increased by head-shaking maneuvers with the vibration in the semicircular canals.

DHI evaluates the handicaps caused by the vestibular disease itself and its effect on quality of life. DHI consists of twenty-five items divided into 3 areas as functional, emotional, and physical. Serbetcioglu et al. has found that DHI has adequate reliability and validity for assessing the effectiveness of vestibular rehabilitation programs in the population in Turkey.^20^ BBS was developed to evaluate the postural control and is widely used in many rehabilitation areas. The fourteen items in the scale assess balance during activities commonly performed in daily functions, including transfers, rotating, and picking up objects from the ground, as well as the balance of static sitting and standing. Sahin et al. showed that the Turkish version of the BBS is a repeatable, reliable, and valid measure of postural control for Turkish-speaking patients.^21^ In our study, pre-treatment and post-treatment results were evaluated by both DHI and BBS in both groups.

Oh et al. stated that the head-shaking maneuver is more effective than the modified symontics maneuver in the treatment of lateral semicircular canal BPPV and that mastoid vibration does not provide additional benefit.^22^ Kong et al. found no statistical difference between cupulolithiasis repositioning maneuver, head-shaking maneuver, and modified Lempert maneuver in the treatment of lateral semicircular canal BPPV.^23^ Kim et al. reported that the advantage of head-shaking maneuver may be its effectiveness in removing the otoliths from the cupula irrespective of their attached side, and easy application in the patients whose lesion side is undetermined.^24^ However, there is no study in the literature regarding the effectiveness of the head-shaking maneuver in the treatment of posterior semicircular canal BPPV. In this study, it was found that the improvement in DHI and BBS in the EM group after head-shaking maneuver was higher than in the group without head-shaking maneuver in the first week.

## Conclusion

As a result of our hypothesis in this study, we believe that in the treatment of posterior semicircular canal BPPV, otoliths adhering to the canal may be mobilized by the vibrations generated by the head-shaking maneuver. We think that mobilization of otoliths adhered to the canal will help increase the effectiveness of EM and improve residual dizziness complaints in the patients.

## Conflicts of interest

The authors declare no conflicts of interest.
